# Barriers to and facilitators of screening for cervical and breast cancer: Experiences of non-adherent women with current or previous drug use

**DOI:** 10.1016/j.pmedr.2024.102641

**Published:** 2024-02-05

**Authors:** Lars Garpenhag, Disa Dahlman

**Affiliations:** a Center for Primary Health Care Research, Department of Clinical Sciences, Clinical Research Center/CRC, Lund University/Region Skåne, Box 503 22, Malmö, Sweden; b Division of Psychiatry, Department of Clinical Sciences Lund, Lund University, BMC I12, 221 84 Lund, Sweden

**Keywords:** Health Services Accessibility, Sweden, Health Equity, Women, Opioid Agonist Treatment, Drug Use, Early Detection of Cancer

## Abstract

**Objective:**

Women with current or previous drug use (WCPDU) have an increased risk of poor cervical and breast cancer outcomes, and low participation in screening for these cancer forms. The aim of this study was to assess self-reported barriers to and facilitators of cervical and breast cancer screening, among WCPDU.

**Methods:**

A survey based on results from a previous qualitative study about WCPDUs experiences of cancer screening was distributed to WCPDU at six opioid agonist treatment (OAT) clinics and one needle exchange program (NEP) in Malmö, Sweden, and through the Drug Users Union in Stockholm, Sweden. Data on barriers to and facilitators of cancer screening reported by 222 non-adherent WCPDU was analyzed by descriptive statistics. The barriers were structured according to an established model of healthcare access.

**Results:**

The most frequently reported barriers to cervical as well as breast cancer screening were non-receipt of screening invitations (36 % and 25 %, respectively), trouble remembering the appointment (27 % and 30 %) and other priorities (26 % and 29 %). Common suggested facilitators for both cervical and breast cancer screening were screening access at, e.g., a NEP or OAT clinic (45 % and 40 %) and practical support to attend the screening appointment (40 % and 38 %).

**Conclusions:**

In contrast to general population women, WCPDU report mainly practical barriers to cervical and breast cancer screening. However, NEP participants frequently reported experience of stigma and poor reception as barriers to screening. Interventions to minimize barriers to screening are crucial to decrease the increased cancer morbidity and mortality among WCPDU.

## Highlights

• Women with current or previous drug use (WCPDU) under-utilize cancer screening.
• WCPDU report mainly practical barriers to cervical and breast cancer screening.
• Practical support and integrated screening are suggested facilitators.
• Screening barriers and facilitators differ between subgroups of WCPDU.
• Screening interventions should be tailored for the needs of WCPDU.

## 1 Introduction

Women with current or previous drug use (WCPDU) form a socially vulnerable group and are at great risk of poor health outcomes, including elevated cervical and breast cancer risks (Reece, 2007, Randall et al., 2011, Kricker et al., 2013, Soccio et al., 2015, Ahlgrén-Rimpiläinen et al., 2020, Dahlman et al., 2021, Dahlman et al., 2021). Early detection and treatment are known to enhance the chances of positive outcomes for cancers in both sites. Thus, the disparities in outcomes – and the fact that WPCDU are known to engage in smoking and risky sexual risk behaviors to a greater degree than women in general (Hwang et al., 2000, Allen et al., 2020, Troberg et al., 2019) – demonstrate the importance of reaching the group with preventive services. Internationally, however, both women who are currently using drugs and those in opioid agonist treatment (OAT) have been found to exhibit lower cervical cancer screening participation than non-drug dependent women (Kricker et al., 2013, Abrams et al., 2012, Haddad et al., 2015). In a recent study on Swedish WCPDU, adherence to national screening guidelines was as low as 29 % for breast cancer, and 41 % for cervical cancer – to be compared with rates of 70 % and 80 % respectively in the general Swedish population (Garpenhag and Dahlman, 2023). This fits well into a greater picture of great levels of unmet somatic healthcare needs and poor healthcare access among people who use drugs (Troberg et al., 2019, Drumm et al., 2003, Heinzerling et al., 2006, Powelson et al., 2014, Artenie et al., 2015, Hyshka et al., 2017, Spithoff et al., 2019, Miller-Lloyd et al., 2020).

People who use drugs are known to face numerous barriers that obstruct healthcare access (Drumm et al., 2003, Hyshka et al., 2017, Miller-Lloyd et al., 2020, Edlin et al., 2005). In the case of cancer screening services specifically, qualitative evidence points to the existence of a range of barriers, from competing needs to stigmatic healthcare reception and practical difficulties (e.g., not receiving an invitation to the appointment), that makes them hard to reach for WCPDU (Garpenhag and Dahlman, 2023). While factors associated with WCPDU participation in colorectal and cervical cancer screening have been studied before (Allar et al., 2023, Murphy et al., 2021, McKnight et al., 2006), the relative weight and importance of different kinds of barriers have not been studied, either in Sweden or elsewhere. The aim of this study was to quantitatively assess the prevalence of experienced barriers to cervical and breast cancer screening among WCPDU, as well as what kinds of support could facilitate screening participation according to WCPDU themselves.

## 2 Methods

A survey was conducted as part of a mixed-methods project on cancer screening participation in Swedish WCPDU (Garpenhag and Dahlman, 2023, Garpenhag and Dahlman, 2023). A subset of survey data on the prevalence of barriers and facilitators reported by participants reporting non-adherence to national screening guidelines was analyzed descriptively.

### 2.1 Setting

The study was set in the cities of Stockholm (∼1.5 million residents) and Malmö (∼350,000 residents), Sweden, the first and third largest urban centers in the country. The Swedish population-based screening programs for cervical and breast cancer are based on guidelines issued by The National Board of Health and Welfare. Women within certain age spans are called up by mail to free-of-charge screening at certain intervals. At the time when the study was designed and conducted, regular screening was recommended to women aged 23–64 for cervical cancer (every third year for women aged 23–49 years and every seventh year for women aged 50–64 years) (The National Board of Health and Welfare, 2015), and aged 40–74 for breast cancer (every second year) (The National Board of Health and Welfare, 2022).

Sweden displays a combination of health services that are universal, comprehensive, and mainly tax-financed, and a traditionally restrictive drug policy (EMCDDA, 2019). Opioid overdose mortality figures are high in an international perspective (EMCDDA, 2020). Addiction services such as OAT and needle exchange programs (NEPs) are covered by the universal health insurance, but availability varies considerably between regions.

### 2.2 Participants

The participants in this study consisted of a sub-sample from a survey study on participation in cervical and breast cancer screening with WCPDU in Stockholm and Malmö, described in detail in Garpenhag and Dahlman (2023). Our definition of WCPDU included women with current or previous illicit drug use, including non-medical use of prescription drugs. All survey participants who reported non-adherence to screening for cervical or/and breast cancer (n = 222) were included in the current study for further analysis.

Recruitment took place at six OAT clinics and the only NEP in Malmö, with the intent to invite all female patients frequenting these clinics at the time of the study. Recruitment of women in Stockholm were carried out in collaboration with the Drug Users’ Union (DUU), a non-profit peer organization. Through the DUU, we reached a broader population of WCPDU, including women with no ongoing contacts with addiction services. For further information regarding the recruitment sites, see Table 1. To be included, a woman had to be at least 23 years old (the minimum age for cervical cancer screening call-up) and either participate in OAT or NEP or be in contact with organizations collaborating with the Stockholm DUU. Persons deemed unable to provide informed consent – due to severe psychiatric conditions, drug influence or poor language skills – were excluded from participation. Clinic and DUU staff were ready to assist participants experiencing problems due to language barriers, dyslexia, or impaired eyesight. All participants provided written informed consent. Upon completion of the questionnaire, participants received grocery store gift cards valid for 100 Swedish crowns (∼U.S. dollar $12 when the study was conducted).

**Table 1:** Sample characteristics of Swedish women with current or previous drug use reporting non-adherence to screening for cervical or breast cancer. Data collection 2021–2022. N = 222.

**Characteristic**	**All** n (%) or years (range; IQR)	**OAT** n (%) or years (range; IQR)	**NEP** n (%) or years (range; IQR)	**None** n (%) or years (range; IQR)
Total n	222	115^a^	48	59

Median age	46 (25–70; 37–54)	43 (25–70; 37–53)	45 (26–65; 35–54)	48 (25–68; 40–56)
Resident in Stockholm	115 (52)	23 (20)	33 (69)	59 (100)
Native language Swedish	196 (88)^1^	100 (87)^1^	38 (79)	58 (98)
Educational level 10 < years	124 (56)^1^	58 (50)^1^	29 (60)	37 (63)
Unstable housing situation^b^	72 (32)^1^	24 (21)^1^	23 (49)	25 (42)
Unstable main source of income^c^	133 (60)^1^	73 (64)	30 (63)^1^	30 (51)

OAT = Opioid agonist treatment. NEP = Needle exchange program. IQR = Interquartile range.

a. Of these, 19 individuals reported participation in both OAT and NEP.

b. Housing situation was recoded to “unstable” if the respondent replied, “unstable housing” or “homeless”.

c. Main source of income was recoded to “unstable” if the respondent replied, “financial support from social services” or “other”.

1. Missing n = 1.

### 2.3 Procedures

Data concerning socioeconomic factors (healthcare contact, age, native language, educational level, housing situation and main source of income), cancer screening participation, and barriers to and facilitators of screening adherence were collected through a self-administered questionnaire, as previously described in greater detail by Garpenhag and Dahlman (2023). In the questionnaire, respondents were asked to indicate all items applicable to them on a list of possible screening barriers, as well as a list of possible facilitators. They were also given the option to add any applicable barriers and facilitators that were not present in the lists, as well as a specification on things they had prioritized over screening, in free text. The parts of the questionnaire that covered screening barriers and facilitators were constructed based on results from a qualitative focus group interview study with women in OAT (Garpenhag and Dahlman, 2023), with items designed to collect data on barriers identified in the qualitative study. We also added one item pertaining to transport costs that was not reported in the qualitative study but has featured prominently in the previous literature on healthcare access and barriers among people who use drugs (Drumm et al., 2003, Miller-Lloyd et al., 2020, Matsuzaki et al., 2018). Because screening for cervical and breast cancer is offered free-of-charge at call up, however, we choose not to include any item on direct costs such as service fees.

Four questions allowed respondents to respond, or specify some of the answers, in free-text form. We coded any free text responses and presented them as categories. The free text specification of the statement ‘I prioritized other things, namely [− − − −]’ were coded as ‘Addiction/drugs’, ‘Other obligations (e.g., work, pets)’, ‘Housing’, ‘Other’ and ‘Not specified/Do not know’. Barriers indicated in free text that were found to correspond with an already existent multiple-choice response were recoded as belonging to the relevant category, while unspecified barriers and barriers not included among the multiple-choices were coded as ‘Other barriers’.

The free text responses to the suggested facilitator ‘Better invitation design – Please explain how’ were coded as ‘Digital invitation’ (including by text message), ‘Invitation via, e.g., OAT or other organization’, ‘Simpler language or more welcoming tone’, ‘Other’, or ‘Not specified’. Suggested facilitators described in free text were recoded as belonging to one of the multiple-choice facilitators when applicable, while unspecified facilitators and facilitators not included in the multiple-choices were coded as ‘Other facilitators’.

### 2.4 Analysis

Data were organized in SPSS Statistics Version 25 (SPSS Statistics Version 25) and analyzed using descriptive statistics. To give a better sense of how and when different barriers could be expected to interfere with screening access, we sort the barriers into four groups, based on their place and function in the healthcare seeking process (Levesque et al., 2013):

A. Barriers pertaining to the perceived need for screening (including conflicting needs and priorities).
B. Barriers pertaining to screening service acceptability (i.e., circumstances that affect the willingness to seek screening services, or attend screening when called up).
C. Barriers pertaining to screening service availability and patients’ abilities to reach them. This category is also referred to as ‘practical barriers’ (e.g., not being aware of the appointment, or having trouble remembering it).
D. Barriers pertaining to monetary (transportation) costs associated with screening.
E. Other (miscellaneous and not specified) barriers.

The distribution of barriers and facilitators in three subgroups of WCPDU – women in OAT (with or without NEP participation), women participating in a NEP (but not OAT), and women participating in neither OAT nor NEP – were descriptively analyzed and is presented as Supplementary material.

### 2.5 Ethical compliance

The study was conducted in accordance with the Declaration of Helsinki 2013 and was approved in advance by the Swedish Ethical Review Authority (file no. 2020-04150).

## 3 Results

### 3.1 Sample characteristics

We received 298 individual survey responses to the original survey. 222 women reported non-adherence to either cervical or breast cancer screening and were thereby included for analysis in this study. The respondents’ median age was 46 years (range 25–70 years), 88 % had Swedish as their native language, 56 % had 10 years or more of education, unstable housing was reported by 32 % and unstable income by 60 % (Table 1). While the full sample was evenly distributed between Malmö (48 %) and Stockholm (52 %), the two sub-samples differed in that the Stockholm sub-sample included a higher proportion of women with a NEP contact, a smaller proportion of women in OAT, and all women lacking both OAT and NEP contacts. Women in OAT had a slightly lower median age than that of the full sample and had lower educational level and less frequently reported unstable housing, compared to NEP participants and women with neither OAT nor NEP contact. Almost all women with neither OAT nor NEP contact (98 %) had Swedish as their native language, and this group reported unstable income less frequently than other sub-groups.

### 3.2 Barriers to screening for cervical and breast cancer

Of the 163 women who reported non-adherence to the recommended screening intervals for cervical cancer, 36 % had missed screening due to not having received an invitation (Table 2). Other frequently reported (by 23–27 % of the sample) barriers to screening were found among those affecting the perception of need for screening (prioritizing other things, time limitations due to addiction), barriers related to screening services acceptability (bad experiences of healthcare services) and barriers related to availability and ability to reach services (simply forgetting).

**Table 2:** Barriers to screening for cervical and breast cancer among Swedish women with current or previous drug use reporting non-adherence to screening. Data collection 2021–2022.

**Characteristic**	**Cervical cancer**n (%)	**Breast cancer**n (%)
Total non-adherent n	163	126

A. Barriers pertaining to the perceived need for screening		
I was afraid to find out that I have cancer	28 (17)^1^	21 (17)
I do not believe that cancer happens to me	13 (8)	11 (9)
I do not care if I have cancer	10 (6)	6 (5)
I do not feel worthy of using healthcare resources	5 (3)	3 (2)
I do not have time, due to my addiction	39 (24)	29 (23)
I prioritized other things	42 (26)*^1^	37 (29)**

B. Barriers pertaining to screening service acceptability		
I have bad experiences of healthcare services	37 (23)	19 (15)
I feel persecuted/stigmatized when I visit healthcare services	29 (18)	20 (16)
I feel that the screening procedure in itself is uncomfortable	30 (18)	23 (18)
I did not want to attend while under the influence^2^	19 (12)	11 (9)

C. Barriers pertaining to screening service availability and patients’ ability to reach them		
I have not received an invitation	58 (36)	32 (25)
I could not comprehend the invitation	6 (4)	4 (3)
I forgot, or missed the appointment by mistake	45 (27)	38 (30)
I did not have the opportunity to prepare (e.g. maintain hygiene)	7 (4)	6 (5)

D. Barriers pertaining to monetary costs associated with screening		
I could not afford transport	14 (9)	5 (4)

E. Other barriers	20 (12)	21 (17)

1. Missing n = 1.

2. Implying that WCPDU were reluctant to attend screening services where they did not feel accepted if they showed up under influence of drugs.

* Addiction/drugs n = 9; Other obligations n = 4; Housing n = 4; Other n = 9; Not specified/do not know n = 16.

** Addiction/drugs n = 7; Other obligations n = 3; Other n = 10; Not specified/do not know n = 17.

The barriers towards breast cancer screening were similar. The most prevalently reported reasons for non-adherence (by 23–30 % of the sample) were simply forgetting, prioritizing other things, not having received an invitation and time limitations due to addiction.

We noted some differences between women in OAT, women participating in NEP, and women with neither OAT nor NEP contact. A larger percentage of women in OAT or NEP than women with neither OAT nor NEP contact reported time limitations due to addiction as a barrier towards cervical cancer screening (26 % OAT, 34 % NEP, 11 % none; Supplementary Table 1) and breast cancer screening (25 % OAT, 38 % NEP, 12 % none; Supplementary Table 2). A notable percentage of women in NEP reported bad experiences of healthcare services (34 %) and stigma in healthcare settings (26 %) as barriers to cervical cancer screening.

### 3.3 Facilitators of screening for cervical and breast cancer

Notable percentages of WCPDU who were non-adherent to cervical cancer screening, suggested that practical support (40 %) or integrated screening (45 %) would be efficient facilitators (Table 3). Other common suggested facilitators were digital invitations (29 %) and screening services catering solely for WCPDU (26 %).

**Table 3:** Facilitators of screening for cervical and breast cancer among Swedish women with current or previous drug use reporting non-adherence to screening. Data collection 2021–2022.

**Characteristic**	**Cervical cancer**n (%)	**Breast cancer**n (%)
Total non-adherent n	163	126

A. Moral and practical support		
Social or psychological support	27 (17)	27 (21)
Practical support, to remember the time and place of appointments	65 (40)	48 (38)

B. Integrated or specialized service delivery		
The opportunity to attend screening at a NEP, OAT clinic or other addiction care service	74 (45)^1^	50 (40)
The opportunity to attend screening at a special clinic for women with drug dependency	43 (26)	32 (25)

C. Enhanced invitational procedures		
Digital invitations, instead of in the mail	47 (29)	27 (21)
Better invitation design	24 (15)*	14 (11)**

D. Other facilitators	20 (12)^1^	19 (15)

1. Missing n = 1.

NEP = Needle exchange program. OAT = Opioid agonist treatment.

* Digital invitation n = 7; Via organization n = 2; Other language n = 3; Other n = 1; Not specified n = 11.

** Digital invitation n = 2; Via organization n = 2; Other language n = 3; Other n = 2; Not specified n = 5.

The most prevalent facilitators to breast cancer screening were also practical support (38 %) and integrated screening (40 %), followed by screening services catering solely for WCPDU (25 %). Practical support was a particularly prevalent suggestion by women in NEP (54 % – OAT 43 %, none 22 %) (Supplementary Table 4).

Women without OAT or NEP contact reported less need for moral and practical support but more need for enhanced invitational procedures. Digital invitations were frequently suggested as a facilitator to cervical cancer screening (38 %; Supplementary Table 3) as well as breast cancer screening (29 %; Supplementary Table 4). Women attending a NEP notably often reported that they thought that integrated or specialized service delivery regarding both cervical cancer screening (71 % and 42 %) and breast cancer screening (75 % and 46 %) would be a facilitator (Supplementary Tables 3 and 4).

## 4 Discussion

While factors associated with WCPDU participation in colorectal and cervical cancer screening have been studied before (Allar et al., 2023, Murphy et al., 2021, McKnight et al., 2006), to the best of our knowledge, there are no previous quantitative studies that specifically investigate WCPDU’s barriers and facilitators to cancer screening, either in Sweden or abroad. In this study we quantitatively assess the prevalence of experienced barriers to cervical and breast cancer screening among WCPDU, as well as what kinds of support would facilitate screening participation according to WCPDU themselves. The study suggest that practical barriers influencing the availability of screening services or the ability to reach them, (e.g., not being aware of the appointment, or having trouble remembering it) are more prevalent than those mainly affecting the perceived need for screening or the acceptability of services (e.g., lack of interest, or experience of stigma in healthcare settings), and a substantial percentage of WCPDU indicates that practical support and integrated screening would facilitate screening participation for them.

The emphasis on practical barriers hampering availability in our findings were somewhat surprising. Previous studies have described the difficulties to prioritize between non-acute healthcare utilization and drug use among people who use drugs (Drumm et al., 2003). Even though a notable percentage of our sample reported that their addiction was a barrier to cervical as well as breast cancer screening (24 % and 23 %, respectively), however, this was not the main barrier to either form of screening. Note that not all participants in our study were actively using drugs, which might explain the somewhat low percentages. In addition, given that stigma and poor experiences of healthcare constitute commonly reported barriers to healthcare seeking in people who use drugs (Miller-Lloyd et al., 2020, Neale et al., 2008, Garpenhag and Dahlman, 2021, Troberg et al., 2022), it could have been expected to be more prominent among the barriers reported by WCPDU in our study. While this could be interpreted as a result of unusually positive attitudes to WCPDU in women’s healthcare, the difference is more probably situational, and dependent on important differences between the attending of screening for cervical and breast cancer and many other kinds of healthcare contacts. To attend cancer screening when called up is to fulfill a social expectation and should bring less worry about being questioned. This is in contrast to WCPDUs’ visits to, e.g., the primary healthcare center, which could be construed as suspicious, for instance as attempted malingering or as part of a scheme to acquire narcotics, issues that are not relevant in the screening situation. Importantly, women participating in a NEP more frequently than others reported bad experiences of healthcare services (34 %) and stigma in healthcare settings (26 %) as barriers to cervical cancer screening. These differences between sub-groups could indicate a hierarchy of stigma, in which women with injection drug use experience worse staff attitudes than other WCPDU. The differences are of clinical importance as previous data from our research group have shown that NEP participants report lower screening adherence than women in OAT and WCPDU with neither OAT nor NEP contact (Garpenhag and Dahlman, 2023).

While we are not aware of any previous studies that assess WCPDU’s suggested cancer screening facilitators, a qualitative study from the U.S. showed that homeless women considered comfort with the healthcare setting and professional taking the sample, to be important facilitators for cervical cancer screening (Wittenberg et al., 2015). In our sample, it was notably common among women attending a NEP to suggest that both cervical and breast cancer screening would be facilitated by integrated services (cervical 71 % and breast 75 %) but also specialized service delivery catering for WCPDU (cervical 42 %, breast 46 %). It could be hypothesized that the more marginalization the individual is subject to, the greater becomes the importance of “one stop-shops” and integrated healthcare services.

In our study, women without OAT or NEP contact reported less need for moral and practical support but more often suggested that enhanced invitational procedures, especially digital invitations, would facilitate cervical as well as breast cancer screening participation. It could be hypothesized that women with no ongoing OAT or NEP contact were less likely to use drugs at the time of the study, and therefore requiring other support than women in OAT and women utilizing NEP services. As our survey did not include questions regarding duration since last time of drug use or main drug, future research is needed to explain the different needs and requests in subgroups of WCPDU.

Some of the self-reported screening barriers that were prevalent in our sample of WCPDU have figured in previous studies on Swedish women in general as well. For instance, feelings of discomfort surrounding the physical screening examination has been found to be a common reason to abstain from cervical cancer screening (Oscarsson et al., 2008) and has been suggested in qualitative studies to be an important reason to abstain from breast cancer screening too (Sterlingova and Lundén, 2018, Norfjord Van Zyl et al., 2018). That other interests or responsibilities conflict with and take priority over the need to participate in breast cancer screening is also a well-known barrier to both cervical and breast cancer screening in Swedish women in general (Oscarsson et al., 2008, Sterlingova and Lundén, 2018, Norfjord Van Zyl et al., 2018). Qualitative results also indicate that practical issues such as the lack of reminders makes participation in breast cancer screening seem a less feasible prospect for certain non-WPCDU women (Sterlingova and Lundén, 2018), and that they can see a need for greater practical flexibility on the part of service providers as well (Norfjord van Zyl et al., 2020).

However, our study also suggests that the barriers that WCPDU encounter differ from those experienced by Swedish women in general in important respects. While travel costs turned out to be an uncommon issue among our respondents, travel time and costs, as well as other non-medical costs, constitute a type of barrier that has featured in several previous studies on participation in breast and cervical cancer screening (Norfjord Van Zyl et al., 2018, Östensson et al., 2015). The discrepancy could be interpreted as a result of several commonly experienced WCPDU barriers appearing earlier in the process of accessing screening. For instance, for women who do not receive the invitation to screening, transportation costs never become an issue. In addition, certain circumstances frequently experienced as barriers by our sample of WCPDU are either closely tied to drug dependence and its consequences (e.g., drug related stigma, unwillingness to frequent services while under the influence) or plausibly attributable to social and financial deprivation (e.g., inability to receive invitations due to unstable housing) and could not be expected to influence screening behaviors in the population at large to any greater degree. Thus, for instance, while mistrust in healthcare services has known associations with breast cancer screening non-attendance in general population samples (Lagerlund et al., 2000), WCPDU have some subjective reasons for feeling such mistrust that they do not share with women who lack a history of drug use.

### 4.1 Study limitations and strengths

A strength of this study is that we have asked WCPDU themselves about what they believe hinders and helps access to screening services, rather than speculated based on previous knowledge about the group and their screening patterns. The differences between subgroups of WCPDU should be taken into account when tailoring interventions aiming at improved cancer screening participation among WCPDU. While our data suggest that on-site or specialized screening, and practical and moral support from healthcare professionals might be efficient for women in continuous contact with OAT clinics or NEPs, other interventions might be necessary in other subgroups of WCPDU.

This study was conducted in a setting where cervical and breast cancer screening is free of charge, does not require a private health insurance, and is actively offered by invitations to women in relevant ages. In addition, both the geographical areas where the study was conducted (Malmö and Stockholm) are characterized by – in a Swedish context – well-functioning NEP and comprehensive OAT coverage, and easy geographical access to screening service providers. The findings might not be transferable to settings displaying a different organization of healthcare services, or to geographical areas in Sweden with less easy-accessible NEP and OAT services.

This study has limitations. The results represent the self-reported beliefs of WCPDUs and is not based on observation of their actual screening behaviors. The small sample size of this study, and the fact that it is not possible to assess the representativity of the sample in Stockholm and Malmö, form further limitations. We know that around 80 % of eligible study participants among OST attenders in Malmö participated, but a lack on data on either the total population of WCPDU or on NEP attendance in Stockholm and Malmö makes it impossible to decide a general response rate. To minimize the risk of selection bias, women who were deemed to be ineligible to participate due to psychiatric problems or substance influence at one occasion were welcome to try again later if circumstances changed. However, it cannot be ruled out that women who met the exclusion criteria were non-adherent to cancer screening to a larger extent that those included in the study. There is thus a risk that we have thereby excluded a particularly marginalized subgroup, something that should be taken into consideration when interpreting the results.

Our study indicates that WCPDU experience a range of barriers to cervical and cancer screening, some of which sets them apart from women without a history of drug use. The main findings, that practical barriers influencing the availability of screening services were the most frequently reported barriers among WCPDU, that WCPDU suggested practical support and integrated screening services, and that there were notable differences between subgroups of WCPDU, have important clinical implications. This is important knowledge that can serve as basis for interventions to increase screening participation in the group. Our findings clearly suggest that interventions should focus on minimizing practical barriers related to, e.g., communication, rather than on improving the content of informational materials about cancer screening. For WCPDU who have an ongoing healthcare contact, practical assistance from healthcare professionals to receive and manage invitations to screening, or in booking and pursuit of screening appointments appear to be possible avenues to increased screening participation. The possibility of on-site screening at OAT clinics and NEPs should also be evaluated. However, neither of these interventions can be used to reach WCPDU without ongoing healthcare contacts. As suggested by the survey respondents themselves, improved invitational procedures – e.g., in the form of phone or e-mail invitations – could make a difference for this group. Other ways to facilitate screening attendance for this sub-group of women could be to create opportunities for drop-in screening sessions, as well as systematized support from the social services.

In efforts to increase cancer screening attendance among WPCDU, we suggest that particular focus should be put on NEP-participating women, as this subgroup frequently reported healthcare stigma and suggestions for specialized screening services catering to WCPDU.

## 5 Conclusion

WCPDU report practical barriers to cervical and breast cancer screening, rather than poor knowledge, experience of stigma or weak interest in screening. Interventions to minimize barriers to cancer screening are crucial to decrease the increased cancer morbidity and mortality among WCPDU. These interventions should be tailored according to the particular needs of WCPDU, with the somewhat different needs in sub-groups of WCPDU taken into consideration.

## Funding source

This work was supported by ALF research grant (“Yngre ALF”), Region Skåne/Lund University, Sweden; a grant from the Swedish Society of Medicine; and research funding granted from the Primary Healthcare Management in Region Skåne (Sweden) to Disa Dahlman. The funding agencies had no role in the design and conduct of the study; in the collection, analysis and interpretation of the data; or in the preparation, review or approval of the manuscript.

## CRediT authorship contribution statement

**Lars Garpenhag:** Writing – review & editing, Writing – original draft, Project administration, Methodology, Investigation, Formal analysis, Conceptualization. **Disa Dahlman:** Writing – review & editing, Writing – original draft, Project administration, Methodology, Investigation, Funding acquisition, Formal analysis, Data curation, Conceptualization.

## Declaration of competing interest

The authors declare that they have no known competing financial interests or personal relationships that could have appeared to influence the work reported in this paper.

## Data Availability

Data will be made available on request.
