# Supplementary material for: Barriers to and facilitators of screening for cervical and breast cancer: Experiences of non-adherent women with current or previous drug use
Source: Prev Med Rep. 2024 Feb 5;39:102641. doi: 10.1016/j.pmedr.2024.102641 (PMC10869932; doi:10.1016/j.pmedr.2024.102641)
Supplement: Supplementary data 1 [file mmc1.docx]

**Supplementary table 1. Barriers to cervical cancer screening in women with current or previous drug use with different healthcare contacts. N=163.**

| **Characteristic** | **OAT**  n (%) | **NEP**  n (%) | **None**  n (%) |
| --- | --- | --- | --- |
|  |  |  |  |
| Total non-compliant n | 80 | 38 | 45 |
|  |  |  |  |
| A. Barriers to perceiving a need for screening |  |  |  |
| I was afraid to find out that I have cancer | 14 (18) | 6 (16) | 8 (18)^1^ |
| I do not believe that cancer happens to me | 7 (9) | 4 (11) | 2 (4) |
| I do not care if I have cancer | 3 (4) | 2 (5) | 5 (11) |
| I do not feel worthy of using healthcare resources | 1 (1) | 1 (2) | 2 (4) |
| I do not have time, due to my addiction | 21 (26) | 13 (34) | 5 (11) |
| I prioritized other things | 21 (26)^1^ | 10 (26) | 12 (27) |
| B. Barriers pertaining to screening service acceptability |  |  |  |
| I have bad experiences of healthcare services | 16 (20) | 13 (34) | 1 (18) |
| I feel persecuted/stigmatized when I visit healthcare services | 14 (18) | 10 (26) | 5 (11) |
| I feel that the screening procedure in itself is uncomfortable | 16 (20) | 8 (21) | 6 (13) |
| I did not want to attend while under the influence | 12 (15) | 3 (8) | 4 (9) |
| C. Barriers pertaining to screening service availability and ability to reach them |  |  |  |
| I have not received an invitation | 23 (29) | 17 (45) | 18 (40) |
| I could not comprehend the invitation | 4 (5) | 1 (3) | 1 (2) |
| I forgot, or missed the appointment by mistake | 22 (28) | 10 (26) | 13 (29) |
| I did not have the opportunity to prepare (e.g. maintain hygiene) | 2 (3) | 2 (5) | 3 (7) |
| D. Barriers pertaining to monetary costs associated with screening |  |  |  |
| I could not afford transport | 6 (8) | 2 (5) | 6 (13) |
| E. Other barriers | 11 (14) | 6 (16) | 3 (7) |
|  |  |  |  |
|  |  |  |  |

1. Missing n=1.

OAT = Opioid agonist treatment.

NEP = Needle exchange program.

**Supplementary table 2. Barriers to breast cancer screening in women with current or previous drug use with different healthcare contacts. N=126.**

| **Characteristic** | **OAT**  n (%) | **NEP**  n (%) | **None**  n (%) |
| --- | --- | --- | --- |
|  |  |  |  |
| Total non-compliant n | 61 | 24 | 41 |
|  |  |  |  |
| A. Barriers to perceiving a need for screening |  |  |  |
| I was afraid to find out that I have cancer | 9 (15) | 5 (21) | 7 (17) |
| I do not believe that cancer happens to me | 6 (10) | 3 (13) | 2 (5) |
| I do not care if I have cancer | 3 (5) | 1 (4) | 2 (5) |
| I do not feel worthy of using healthcare resources | 1 (2) | 1 (4) | 1 (2) |
| I do not have time, due to my addiction | 15 (25) | 9 (38) | 5 (12) |
| I prioritized other things | 18 (30) | 7 (29) | 12 (29) |
| B. Barriers pertaining to screening service acceptability |  |  |  |
| I have bad experiences of healthcare services | 11 (18) | 5 (21) | 3 (7) |
| I feel persecuted/stigmatized when I visit healthcare services | 10 (16) | 5 (21) | 5 (12) |
| I feel that the screening procedure in itself is uncomfortable | 16 (26) | 3 (13) | 4 (10) |
| I did not want to attend while under the influence | 7 (12) | 1 (4) | 3 (7) |
| C. Barriers pertaining to screening service availability and ability to reach them |  |  |  |
| I have not received an invitation | 9 (15) | 10 (42) | 13 (32) |
| I could not comprehend the invitation | 2 (3) | 1 (4) | 1 (2) |
| I forgot, or missed the appointment by mistake | 23 (38) | 7 (29) | 8 (20) |
| I did not have the opportunity to prepare (e.g. maintain hygiene) | 1 (2) | 3 (13) | 2 (5) |
| D. Barriers pertaining to monetary costs associated with screening |  |  |  |
| I could not afford transport | 0 | 1 (4) | 4 (10) |
| E. Other barriers | 7 (12) | 8 (33) | 6 (15) |
|  |  |  |  |
|  |  |  |  |

1. Missing n=1.

OAT = Opioid agonist treatment.

NEP = Needle exchange program.

**Supplementary table 3. Facilitators of cervical cancer screening in women with current or previous drug use with different healthcare contacts. N=163.**

| **Characteristic** | **OAT**  n (%) | **NEP**  n (%) | **None**  n (%) |
| --- | --- | --- | --- |
|  |  |  |  |
| Total non-compliant n | 80 | 38 | 45 |
|  |  |  |  |
| A. Moral and practical support |  |  |  |
| Social or psychological support | 16 (20) | 7 (18) | 4 (9) |
| Practical support, to remember the time and place of appointments | 34 (43) | 16 (42) | 15 (33) |
| B. Integrated or specialized service delivery |  |  |  |
| The opportunity to attend screening at a NEP, OAT clinic or other addiction care service | 40 (50) | 27 (71) | 7 (16)^1^ |
| The opportunity to attend screening at a special clinic for women with drug dependency | 17 (21) | 16 (42) | 10 (22) |
| C. Enhanced invitational procedures |  |  |  |
| Digital invitations, instead of in the mail | 21 (26) | 9 (24) | 17 (38) |
| Better invitation design | 11 (14) | 4 (11) | 9 (20) |
| D. Other facilitators | 7 (9)^1^ | 7 (18) | 6 (13) |
|  |  |  |  |

1. Missing n=1.

OAT = Opioid agonist treatment.

NEP = Needle exchange program.

**Supplementary table 4. Facilitators of breast cancer screening in women with current or previous drug use with different healthcare contacts. N=126.**

| **Characteristic** | **OAT**  n (%) | **NEP**  n (%) | **None**  n (%) |
| --- | --- | --- | --- |
|  |  |  |  |
| Total non-compliant n | 61 | 24 | 41 |
|  |  |  |  |
| A. Moral and practical support |  |  |  |
| Social or psychological support | 14 (23) | 5 (21) | 8 (20) |
| Practical support, to remember the time and place of appointments | 26 (43) | 13 (54) | 9 (22) |
| B. Integrated or specialized service delivery |  |  |  |
| The opportunity to attend screening at a NEP, OAT clinic or other addiction care service | 27 (44) | 18 (75) | 5 (12) |
| The opportunity to attend screening at a special clinic for women with drug dependency | 11 (18) | 11 (46) | 10 (24) |
| C. Enhanced invitational procedures |  |  |  |
| Digital invitations, instead of in the mail | 10 (16) | 5 (21) | 12 (29) |
| Better invitation design | 5 (8) | 5 (21) | 4 (10) |
| D. Other facilitators | 6 (10) | 4 (17) | 9 (22) |
|  |  |  |  |

1. Missing n=1.

OAT = Opioid agonist treatment.

NEP = Needle exchange program.
